# Academic self-efficacy: from educational theory to instructional practice

**DOI:** 10.1007/s40037-012-0012-5

**Published:** 2012-04-11

**Authors:** Anthony R. Artino

**Affiliations:** Preventive Medicine & Biometrics, Medicine, Uniformed Services University of the Health Sciences, 4301 Jones Bridge Road, Bethesda, MD 20814 USA

**Keywords:** Self-efficacy, Motivation, Academic achievement, Knowledge and skill acquisition, Medical education, Calibration

## Abstract

Self-efficacy is a personal belief in one’s capability to organize and execute courses of action required to attain designated types of performances. Often described as task-specific self-confidence, self-efficacy has been a key component in theories of motivation and learning in varied contexts. Furthermore, over the last 34 years, educational researchers from diverse fields of inquiry have used the notion of self-efficacy to predict and explain a wide range of human functioning, from athletic skill to academic achievement. This article is not a systematic review of the empirical research on self-efficacy; instead, its purpose is to describe the nature and structure of self-efficacy and provide a brief overview of several instructional implications for medical education. In doing so, this article is meant to encourage medical educators to consider and explicitly address their students’ academic self-efficacy beliefs in an effort to provide more engaging and effective instruction.

Since the publication of Albert Bandura’s seminal article entitled ‘Self-Efficacy: Toward a Unifying Theory of Behavioral Change,’[1] countless researchers in the social and behavioral sciences have used self-efficacy to predict and explain a wide range of human functioning. Additionally, over the last 34 years, the tenets of self-efficacy have been extended far beyond the bounds of psychology, reaching fields as diverse as health, medicine, social and political change, psychopathology, athletics, business, and international affairs [2, 3]. During the last decade, research on student self-efficacy has received increasing attention in the area of academic motivation and achievement [4–7]. The purpose of this article is to describe the nature and structure of self-efficacy, a key component of social-cognitive theory, and to provide a brief overview of several potential instructional implications for medical education. Ultimately, by explicating Bandura’s theory of self-efficacy, this article encourages medical educators to consider and explicitly address their students’ academic self-efficacy beliefs in an effort to provide more engaging and effective instruction.

## The nature and structure of self-efficacy

The self-efficacy component of Albert Bandura’s social-cognitive theory is believed by many scholars to be a critically important theoretical contribution to the study of academic achievement, motivation, and learning [2, 3, 8]. In his influential book on the topic, Bandura [9] summarized the importance of self-efficacy in the following way:

‘People make causal contributions to their own psychosocial functioning through mechanisms of personal agency. Among the mechanisms of agency, none is more central or pervasive than beliefs of personal efficacy. Unless people believe they can produce desired effects by their actions, they have little incentive to act. Efficacy belief, therefore, is a major basis of action. People guide their lives by their beliefs of personal efficacy.’

According to Bandura [1, 9, 10], self-efficacy beliefs lie at the core of human functioning. It is not enough for individuals to possess the requisite knowledge and skills to perform a task; they also must have the conviction that they can successfully perform the required behavior(s) under typical and, importantly, under challenging circumstances. Effective functioning, then, requires skills and efficacy beliefs to execute them appropriately—two components that develop jointly as individuals grow and learn. Moreover, these two components of successful human functioning act upon one another in reciprocal fashion, what Bandura [9] has called ‘reciprocal causation,’ where the functioning of one component depends, in part, upon the functioning of the other.

### Self-efficacy defined

Bandura [10] defined self-efficacy as: ‘People’s judgments of their capabilities to organize and execute courses of action required to attain designated types of performances.’ Two important aspects of this definition warrant further explanation. First, self-efficacy is a *belief* about one’s capability, and as such, does not necessarily match one’s actual capability in a specific domain. In fact, research findings have suggested that most individuals actually overestimate their academic capabilities [2, 9]. Bandura [10] argued, however, that the most useful efficacy judgments are those that slightly exceed one’s actual capabilities, as this modest overestimation can actually increase effort and persistence during difficult times. A second important aspect of Bandura’s definition of self-efficacy is the idea that individuals make use of their efficacy judgments in reference to some *goal* (‘attain designated types of performances’), which reflects both the task- and situation-specific nature of efficacy beliefs. This aspect of self-efficacy stands in contrast to other, more general measures of expectancy, such as self-concept and self-perceptions of competence which, although they may be domain specific, tend to be more global self-perceptions [2].

### Self-efficacy influences on human functioning

Bandura [1] hypothesized that self-efficacy affects an individual’s choice of activities, effort, and persistence. People who have low self-efficacy for accomplishing a specific task may avoid it, while those who believe they are capable are more likely to participate. Moreover, individuals who feel efficacious are hypothesized to expend more effort and persist longer in the face of difficulties than those who are unsure of their capabilities [1, 9]. The tendency for efficacious people to ‘expend more effort and persist longer’ is of particular importance because most personal success requires persistent effort. As such, low self-efficacy becomes a self-limiting process. In order to succeed, then, individuals need a strong sense of task-specific self-efficacy, tied together with resilience to meet the unavoidable obstacles of life [9].

### Sources of self-efficacy

Self-efficacy theory postulates that people acquire information to evaluate efficacy beliefs from four primary sources: (a) enactive mastery experiences (actual performances); (b) observation of others (vicarious experiences); (c) forms of persuasion, both verbal and otherwise; and (d) ‘physiological and affective states from which people partly judge their capableness, strength, and vulnerability to dysfunction’ [9]. Of these four information sources, research has shown that enactive mastery experiences are the most influential source of efficacy information because they provide the most direct, authentic evidence that an individual can gather the personal resources necessary to succeed [1, 9]. As one might expect, past successes raise efficacy beliefs, while repeated failures, in general, lower them [1]. However, the influence of performance successes and failures is a bit more complex than this. For example, ‘after strong efficacy expectations are developed through repeated success, the negative impact of occasional failures is likely to be reduced’ [1]. Thus, the effects of failure on personal efficacy really depend on the strength of individuals’ existing efficacy beliefs, as well as the timing of failures with respect to the totality of their performance experiences. In other words, later failures may not negatively impact efficacy beliefs to the same extent as earlier failures might.

While experienced mastery has been shown to produce the most powerful influence on efficacy beliefs, individuals can also learn by observing the successes and failures of others. According to Bandura [1, 9], so-called vicarious experiences can generate efficacy beliefs in observers that they too can attain success through persistence and effort. However, such vicarious experiences, which rely on social comparisons and modeling, are postulated to be less dependable sources of information about one’s own capabilities than is experienced mastery. As such, efficacy beliefs induced solely by observation and modeling of others tend to be weaker and more susceptible to change [1].

A third source of efficacy information comes from verbal persuasion from others. Such social persuasion is widely used in academic settings to help students believe that they can in fact cope with difficult situations. In the words of Bandura: [9] ‘verbal persuasion alone may be limited in its power to create enduring increases in perceived efficacy, but it can bolster self-change if the positive appraisal is within realistic bounds.’ On the other hand, overly optimistic persuasive comments tend to be ineffective, particularly if the individual being persuaded ultimately fails—a result that acts to discredit the persuader and undermine the recipient’s efficacy beliefs [9].

The fourth and final source of efficacy information comes from one’s own physiological and emotional feedback during performance, particularly that involving physical activity. In particular, individuals interpret stress reactions (e.g., increased heart rate, sweating, hyperventilation, and feelings of anxiety and fear) during demanding tasks as signs of vulnerability [9]. Because excessive physiological and emotional arousal can often negatively impact performance, individuals tend to expect success, to a greater extent, when they are *not* overcome by stress reactions than if they are ‘tense and viscerally agitated’ [1]. Unfortunately, fear reactions tend to generate further thoughts of impending danger, thereby significantly elevating an individual’s anxiety level far beyond what may be warranted by the actual situation. Ultimately, information conveyed by physiological reactions is cognitively assessed by individuals and can positively or negatively influence efficacy beliefs, depending on the level of arousal and a person’s cognitive appraisal [9].

### Measuring self-efficacy: domain specificity

An important aspect of self-efficacy is its domain specificity. That is, people judge their capability depending on the particular domain of functioning [11]. Personal efficacy, then, is not a general disposition void of context, but rather a self-judgment that is specific to the activity domain. As such, high self-efficacy in one domain does not necessarily mean high efficacy in another. For example, a medical student may have high efficacy for taking a history or conducting a physical exam, but that same student may have low self-efficacy for understanding biochemistry. Therefore, to achieve predictive power, measures of perceived self-efficacy should be ‘tailored to domains of functioning and must represent gradations of task demands within those domains’ [9].

In educational research, perceived self-efficacy is often measured using self-report surveys that ask participants to rate the strength of their belief in their ability to execute the requisite activities [11]. In many cases, however, educational researchers have mis-measured self-efficacy due, in a large part, to their misunderstanding of the construct [2, 9, 11]. As Pajares [2] pointed out: ‘Because judgments of self-efficacy are task and domain specific, global or inappropriately defined self-efficacy assessments weaken effects.’ Therefore, a researcher attempting to predict or explain an academic outcome, for instance, is more likely to find a strong relationship between self-efficacy and the outcome of interest if the efficacy scale follows two theoretical guidelines: (a) it assesses *specific aspects* of the task and (b) the specificity *corresponds* to the characteristics of the task being assessed and the domain of functioning being analyzed. In Bandura’s [1] words: ‘this requires clear definition of the activity domain of interest and a good conceptual analysis of its different facets, the types of capabilities it calls upon, and the range of situations in which these capabilities might be applied.’ Thus ‘omnibus measures’ of general, contextless dispositions have relatively weak predictive power, whereas domain-linked measures of perceived efficacy have been shown empirically to be good predictors of numerous outcomes, including such diverse criteria as academic performance, pain tolerance, proneness to anxiety, and political participation [9, 12].

Although it is clear that task and domain-specific measures of perceived efficacy have greater predictive power than global measures of the construct, Bandura [9] warned that it is incorrect to believe that self-efficacy is concerned solely with ‘specific behaviors in specific situations.’ In his words: ‘domain particularity does not necessarily mean behavioral specificity’ [9]. In fact, Bandura distinguished among three levels of generality of assessment. The most specific level measures self-efficacy for a particular accomplishment under a narrowly defined set of conditions. The next level measures perceived efficacy for a class of performances within the same domain and under similar conditions. Finally, the most general level ‘measures belief in personal efficacy without specifying the activities or the conditions sharing common properties’ [9]. As discussed before, however, undifferentiated, contextless measures of perceived self-efficacy have meagre predictive power. Thus, Bandura [9] advised: ‘the optimal level of generality at which self-efficacy is assessed varies depending on what one seeks to predict and the degree of foreknowledge of the situational demands.’

## Self-efficacy in medical education: instructional implications

Since Bandura’s seminal article on self-efficacy [1], there has been an accumulation of research evidence supporting the positive links between students’ academic efficacy and their achievement. Specifically, the evidence has shown that students with high self-efficacy in various academic domains choose to engage in tasks that foster the development of their knowledge, skills, and abilities in those areas; exert effort in the face of difficulty; and persist longer at challenging tasks [7, 8]. Furthermore, besides the positive influence that self-efficacy appears to have on the *quantity* of effort, there is evidence that students high in academic efficacy differ in terms of the *quality* of their effort, using more deep cognitive and metacognitive processing strategies than their counterparts with weaker efficacy beliefs [13].

While educators are understandably concerned about teaching students knowledge and skills, results from more than 30 years of self-efficacy research have made it clear that simply possessing knowledge and skills does not ensure that learners will be motivated to apply them [8]. Instead, students need both ‘the skill and the will’ to successfully function within different domains and under a variety of circumstances [13]. In fact, much of the research suggests that students’ perceptions of confidence (i.e., their self-efficacy beliefs) may more accurately predict their motivation and future academic choices than actual competence. Therefore, Bandura and others have suggested that teachers would do well to implement instructional practices that not only foster knowledge and skill attainment, but also promote the development of the necessary accompanying confidence [2, 9, 12]. At the same time, efficacy scholars caution that attempting to build positive efficacy beliefs through programmes that overemphasize verbal persuasion methods is unlikely to be successful [7–9, 12]. Instead, educators should focus their efforts primarily on providing students with authentic mastery experiences. Clearly, instructional strategies focused on providing students with opportunities for performance success align well with Bandura’s [1, 9, 10] emphasis on enactive attainment as the most influential source of self-efficacy information.

With a sound understanding of academic self-efficacy, educators are well positioned to develop and implement effective instructional strategies. Several specific examples of how medical educators might apply the tenets of academic self-efficacy into instructional practice are provided below.

1. *Help students set clear and specific goals*. Research has shown that when students set clear and specific goals, or are given a reasonable goal by a teacher, they are more motivated to perform than students who are given no goals or who are simply told to try their best [14]. According to Bandura [9], students who set a goal are likely to experience an initial sense of self-efficacy for achieving the goal and are also apt to make a commitment to persist. As students work at the task, ‘they engage in activities that they believe will lead to goal attainment: attend to instruction, rehearse information to be remembered, expend effort, and persist’ [8]. Thus, students’ self-efficacy is validated as they observe goal progress and see that they are becoming more knowledgeable and/or skilful.

Research has also shown that individuals with high self-efficacy tend to set loftier goals than do individuals with lower self-efficacy [15]. What is more, those with higher self-efficacy also tend to be more ‘committed to assigned goals, find and use better task strategies to attain the goals, and respond more positively to negative feedback than do people with low self-efficacy’ [15]. Thus, the relationship between goal setting and self-efficacy is reciprocal: goal setting helps to grow self-efficacy, while increased self-efficacy improves the quality of later goals. Of course, in medical education, students are not always free to choose their own goals. Nonetheless, instructors can help students carefully construct clear and specific goals. This might involve, for instance, providing students with examples of well-articulated goals for a clerkship, asking them to develop their own clear and specific goals, and then reviewing these goals with students throughout the clerkship [16].

2. *Encourage the use of challenging and proximal goals*. Goals should be challenging but not outside the range of students’ capabilities [15]. Difficult but achievable goals give students the opportunity to put forth effort and obtain feedback as they make progress toward goal completion. Goals that are too far beyond students’ knowledge or skill level will likely lead to frustration and may actually degrade efficacy beliefs [7]. Moreover, research has shown that proximal goals tend to provide better efficacy information for students than do distant goals, because students can judge progress toward goal achievement with the former better than with the latter [8, 14, 15].
3. *Provide honest, explicit feedback to increase students’ efficacy beliefs*. Honest, explicit feedback, in the form of verbal persuasion and/or rewards that are given contingent upon performance, provides efficacy information to learners and encourages their continued movement toward goal attainment [17, 18]. Praising students non-contingently can be detrimental in that students do not get useful feedback on the development of their actual knowledge and skills. Without explicit feedback on the growth of their knowledge and skills, students will likely have a difficult time trying to change or regulate their behavior. For example, praising students indiscriminately for performing a task, regardless of how *well* they perform, can lead students to think they are good at a task when really they are not [7].

Although a detailed account of the characteristics of effective feedback is beyond the scope of the present article, much has been written on the topic [17, 18]. For example, based on their systematic review of the empirical literature on feedback and its influence on learning, Hattie and Timperley [17] suggest that one of the primary purposes of feedback is to help students enhance their self-efficacy beliefs by correctly attributing their successes and failures. In doing do, students are encouraged to direct their attention back to specifics of the task at hand. Moreover, by developing their self-efficacy beliefs, students are apt to invest more effort and commitment to the current task and future attempts.

4. *Facilitate accurate calibration of self*-*efficacy*. For self-efficacy beliefs to have a positive impact on learning and performance, individuals must have realistic or accurate perceptions of their ability for a given task [19]. Calibration is a measure of the difference between confidence in performance of specific activities (i.e., self-efficacy) and actual performance [19]. Unfortunately, students do not always accurately estimate their self-efficacy. In fact, as described earlier in this article, most individuals tend to overestimate their capabilities [20]. Nonetheless, accurate calibration of self-efficacy is pedagogically important because poor calibration can work against the benefits of high self-efficacy. For example, a medical student who has overestimated his self-efficacy may possess high confidence in his ability to draw blood after learning the skill, but yet he may fail to correctly draw blood from a patient in a clinical setting. And while Bandura [9] proposed that feeling slightly overconfident is adaptive, in the clinical setting it is clear that overestimated self-efficacy beliefs could result in an unsafe environment for patients. On the other hand, a medical student who has underestimated his self-efficacy may possess low confidence in his ability to suture a laceration, and because of this may be unmotivated to practice the skill or persist in the face of difficulties during a practice session.

Several techniques have been suggested for helping students accurately calibrate their self-efficacy beliefs. The most important of these has already been discussed: feedback. Feedback not only helps students grow their self-efficacy beliefs, as described above, but it can play a prominent role in calibration. In particular, immediate feedback that encourages students to shift their focus from actual performance to performance monitoring and evaluation is thought to be particularly effective in improving self-efficacy calibration [17, 19]. Related to this so-called *calibration feedback* is the notion of explicitly teaching students how to use metacognitive study strategies, such as self-testing and self-monitoring, to improve their judgments of knowing [19]. The importance of metacognitive learning strategies has long been acknowledged by educational researchers. In fact, in an early comprehensive review of 179 review articles, Wang and colleagues [21] found that students’ use of metacognitive study strategies was the single most important determinant of school learning. More recently, in a practice guide developed by the Institute of Education Sciences in the United States, metacognitive strategies such as self-testing and self-monitoring were listed as two of the seven most effective behaviors for improving learner retention and deep understanding [22].

5. *Use peer modeling to build self*-*efficacy*. Next to experienced mastery, vicarious experiences have been shown to be powerful influences on efficacy beliefs. As Schunk [8] described: ‘observing others succeed can convey to observers that they too are capable and can motivate them to attempt the task.’ From an instructional perspective, teachers can use other students as models to demonstrate how to successfully complete a learning task (e.g., by asking a medical student to perform a clinical procedure for other trainees to observe). However, teachers need to be aware that not all models are equally effective. In general, models have a greater influence on observers’ self-efficacy when they are perceived as competent, similar, credible, and enthusiastic [10]. With these characteristics in mind, teachers can better enhance learner efficacy by (a) having peer models display skills correctly (competence); (b) using peer models of equal or slightly greater competence than observers (perceived similarity); (c) ensuring that peer models act consistently with the behaviors they model (credibility); and (d) choosing peer models who show interest and enthusiasm, which also holds true for teachers who, themselves, can be informative models.

## Conclusions

The self-efficacy component of Bandura’s social-cognitive theory has had a profound impact on the study of motivation and achievement in academic settings. Results from a meta-analysis of more than 100 empirical studies conducted over the last 20 years found that of nine commonly researched psychosocial constructs, academic self-efficacy was the strongest single predictor of college students’ academic achievement and performance [23]. It seems, then, that cultivating students’ academic self-efficacy is a worthwhile goal for any educator. Bandura [9] made this very argument when he stated: ‘the major goal of formal education should be to equip students with the intellectual tools, efficacy beliefs, and intrinsic interests needed to educate themselves in a variety of pursuits throughout their lifetime.’ As information technologies continue to revolutionize teaching, learning, and the practice of medicine [24], it seems likely that strong, resilient efficacy beliefs will become even more critical for individuals—particularly health care professionals—as they attempt to exercise control over their own learning in progressively more independent, technology-mediated learning environments.

## Essentials

- Self-efficacy is a personal belief in one’s capability to organize and execute courses of action required to attain designated types of performances.
- Self-efficacy is hypothesized to affect an individual’s choice of activities, effort, and persistence across a wide range of human functioning.
- Self-efficacy does not equate to a general confidence in one’s competence; instead, it is more task and situation specific. Individuals develop self-efficacy beliefs in relation to specific goals, such as the ability to accurately assess heart sounds in a patient.
- Medical educators can implement instructional practices that foster both knowledge and skill attainment, along with the development of the necessary accompanying self-efficacy.
- Instructional practices designed to develop students’ self-efficacy beliefs and improve learning include the following: encourage students to set clear, specific, and challenging proximal goals; provide students with honest and explicit feedback; facilitate accurate calibration of self-efficacy; and use peer modeling.
